# Association between ovalocytosis and *Plasmodium* infection: a systematic review and meta-analysis

**DOI:** 10.1038/s41598-023-34170-3

**Published:** 2023-05-03

**Authors:** Kwuntida Uthaisar Kotepui, Aongart Mahittikorn, Frederick Ramirez Masangkay, Manas Kotepui

**Affiliations:** 1 Medical Technology, School of Allied Health Sciences, Walailak University, Tha Sala, Nakhon Si Thammarat, Thailand; 2 Department of Protozoology, Faculty of Tropical Medicine, Mahidol University, Bangkok, Thailand; 3 Department of Medical Technology, University of Santo Tomas, Manila, Philippines

**Keywords:** Haematological diseases, Malaria

## Abstract

Reports of an association between ovalocytosis and protection against *Plasmodium* infection are inconsistent. Therefore, we aimed to synthesise the overall evidence of the association between ovalocytosis and malaria infection using a meta-analysis approach. The systematic review protocol was registered with PROSPERO (CRD42023393778). A systematic literature search of the MEDLINE, Embase, Scopus, PubMed, Ovid, and ProQuest databases, from inception to 30 December 2022, was performed to retrieve studies documenting the association between ovalocytosis and *Plasmodium* infection. The quality of the included studies was assessed using the Newcastle–Ottawa Scale. Data synthesis included a narrative synthesis and a meta-analysis to calculate the pooled effect estimate (log odds ratios [ORs]) and 95% confidence intervals (CIs) using the random-effects model. Our database search retrieved 905 articles, 16 of which were included for data synthesis. Qualitative synthesis revealed that over half of the studies showed no association between ovalocytosis and malaria infections or severity. Furthermore, our meta-analysis demonstrated no association between ovalocytosis and *Plasmodium* infection (*P* = 0.81, log OR = 0.06, 95% CI − 0.44 to 0.19, I^2^: 86.20%; 11 studies). In conclusion, the meta-analysis results demonstrated no association between ovalocytosis and *Plasmodium* infection. Hence, the role of ovalocytosis in relation to protection against *Plasmodium* infection or disease severity should be further investigated in larger prospective studies.

## Introduction

Malaria is caused by at least six *Plasmodium* species, including *Plasmodium falciparum*, *P. vivax*, *P. malariae*, *P. ovale curtisi*, *P. ovale wallikeri*, and *P. knowlesi*^1,2^. The parasite is transmitted between humans via the bite of the female *Anopheles* mosquito^3^. The World Health Organization estimated that there were 247 million malaria cases in 84 malaria-endemic countries in 2021, resulting in 619,000 deaths^4^. *Plasmodium* infection requires interaction with several protein receptors in the membrane of the red blood cell (RBC). The most important of these is *P. falciparum* erythrocyte membrane protein 1, which mediates the interaction of parasite ligands on the RBC surface^5^. Other important malaria parasite ligand/RBC receptors involved in RBC invasion are band 3, glycophorin A, glycophorin B, glycophorin C, Duffy antigen receptor for chemokines and semaphorin 7A^6^.

Southeast Asian ovalocytosis (SAO) is an RBC disorder caused by a 27-basepair deletion in the band 3 protein gene and is characterised by the presence of macro-ovalocytes and ovalostomatocytes on a blood smear^7,8^. Band 3 is a member of the Solute Carrier 4 family of bicarbonate transporters. It is the predominant glycoprotein of the RBC membrane, where it acts as an electroneutral anion exchanger^9,10^. A mutation in or decreased expression of the Band 3 gene has been associated with changes in RBC morphology, membrane instability, and blebbing^11^. In vitro studies have shown that SAO RBCs are relatively resistant to invasion by certain *P. falciparum* isolates^12,13^. Furthermore, an in vitro study recently revealed that *P. vivax* field isolates invaded SAO reticulocytes 67–71% less frequently than non-SAO reticulocytes^14^. Additionally, a clinical study demonstrated an association between SAO and protection against clinical infections of *P. vivax* and severe *P. falciparum* malaria^15^. Although ovalocytosis, particularly SAO, has been reported to confer a protective effect against malaria infection and severity, there are some inconsistencies in the association between ovalocytosis and malaria, with some studies reporting no such association^16,17^. Therefore, our systematic review and meta-analysis aimed to collate and pool the evidence of ovalocytosis-associated malaria infection and severity.

## Methods

### Reporting guidelines and registration

This systematic review and meta-analysis followed the Preferred Reporting Items for Systematic Reviews and Meta-analyses (PRISMA) guidelines^18^. The systematic review protocol was registered with PROSPERO (CRD42023393778).

### Data sources and searches

A systematic search of the MEDLINE, Embase, Scopus, PubMed, Ovid, and ProQuest databases was performed from database inception to 30 December 2022 to retrieve studies reporting cases of malaria in patients with ovalocytosis. The search strategy combined search terms with Boolean operators (AND, OR) as follows: (Elliptocyt* OR Ovalocyt*) AND (malaria OR *Plasmodium* OR ‘Remittent Fever’ OR ‘Marsh Fever’ OR Paludism). The searches were performed in the English language without limitations on the publication date. A search in Google Scholar was also performed on 30 December 2022 to expand the search and ensure that relevant studies were not missed in the search of the main databases. Table S1 presents the details of the literature retrieved from each database.

### Eligibility criteria

The population, exposure, outcome approach^19^ was applied to retrieve eligible studies as follows: (i) P: participants with and without ovalocytosis, (ii) E: malaria and (iii) O: odds/risk of malarial infection. Microscopy, rapid diagnostic tests (RDTs), or molecular methods can be used to detect *Plasmodium* infection. The following articles were excluded with reasons: reviews, in vitro studies, case reports or series, conference abstracts without full reports, books or book chapters, letters or news articles, articles where the outcomes of interest could not be extracted and articles for which full texts were unavailable.

### Study selection and data extraction

The studies retrieved from the database searches were managed using EndNote version 20.0 (Clarivate, London, UK) reference management software. The study selection began by removing duplicate studies, and the titles and abstracts of the remaining studies were screened. Those with irrelevant titles and abstracts were removed, and then the full-text versions of potentially relevant studies were examined. Studies were included based on pre-specified eligibility criteria, and ineligible studies were excluded with specific reasons. Finally, the following data were extracted and input into a data sheet before synthesis: first author, year of publication, study design, country, participants’ characteristics, *Plasmodium* species, age range (years) of patients, characteristics of ovalocytosis, method for investigating ovalocytosis (microscopy, RDT or molecular methods) and method for *Plasmodium* detection. Study selection and data extraction were independently conducted by three authors (MK, KUK and AM) and any discrepancies or disagreements between two authors were resolved through consensus.

### Risk of bias assessment

Two authors (MK and KUK) independently assessed the risk of bias of the included studies using the Newcastle–Ottawa Scale (NOS)^20^. This tool was designed to evaluate the quality of non-randomised studies from three broad perspectives: study group selection, study group comparability and identification of the exposure or outcome of interest for case–control or cohort studies, respectively. Each item received 1 star in the categories of selection and outcome. For each item in the comparability category, a maximum of 2 stars was awarded.

### Data synthesis

Both qualitative and quantitative data were synthesised. The qualitative synthesis involved a narrative synthesis of the outcomes of interest from the included studies. The quantitative synthesis involved a meta-analysis approach to pool the outcomes of interest from the included studies.

To synthesise the pooled log odds ratio (OR) and 95% confidence interval (CI), data in 2 × 2 tables (Table S2) were computed using the random-effects model as described by DerSimonian and Laird^21^. The consistency index (I^2^ statistics) and Chi-square (Q) test were used to assess the significance and levels of heterogeneity among the included studies. I^2^ values of < 25%, 25%–75% and > 75% were interpreted as low, moderate and high levels of heterogeneity, respectively^22^. Meanwhile, a *P* value of 0.1 for the Q statistic was considered an indication of significant heterogeneity among the included studies, as described previously^23^. If the heterogeneity of effect estimates among the included studies was observed, with a *P* value < 0.1 for the Q statistic or an I^2^ value > 25%, the pooled effect estimate was computed using the random-effects model. However, if there was no significant heterogeneity of effect estimates among the included studies, with a *P* value > 0.1 for the Q statistic or an I^2^ value < 25%, the pooled effect estimate was computed using the fixed-effects model.

The publication bias was assessed by visualisation of the funnel plot asymmetry and further validated using Egger’s test^24^. The trim-and-fill method was used to correct for publication bias^25^, if any. Meta-regression analysis based on study design, country, participants’ characteristics, *Plasmodium* species, method used to investigate ovalocytosis, and method for malaria detection was performed to assess the impact of these parameters on the pooled effect estimate. If the meta-regression analysis showed a significant impact of covariates on the pooled effect estimate, a subgroup analysis was conducted to determine the difference in the pooled effect estimate between subgroups. The leave-one-out sensitivity analysis was performed to test whether a single study affected the overall pooled estimate. All analyses were performed using the meta command in Stata v17 software (StataCorp LLC, College Station, TX).

## Results

### Search results

**Figure 1:**
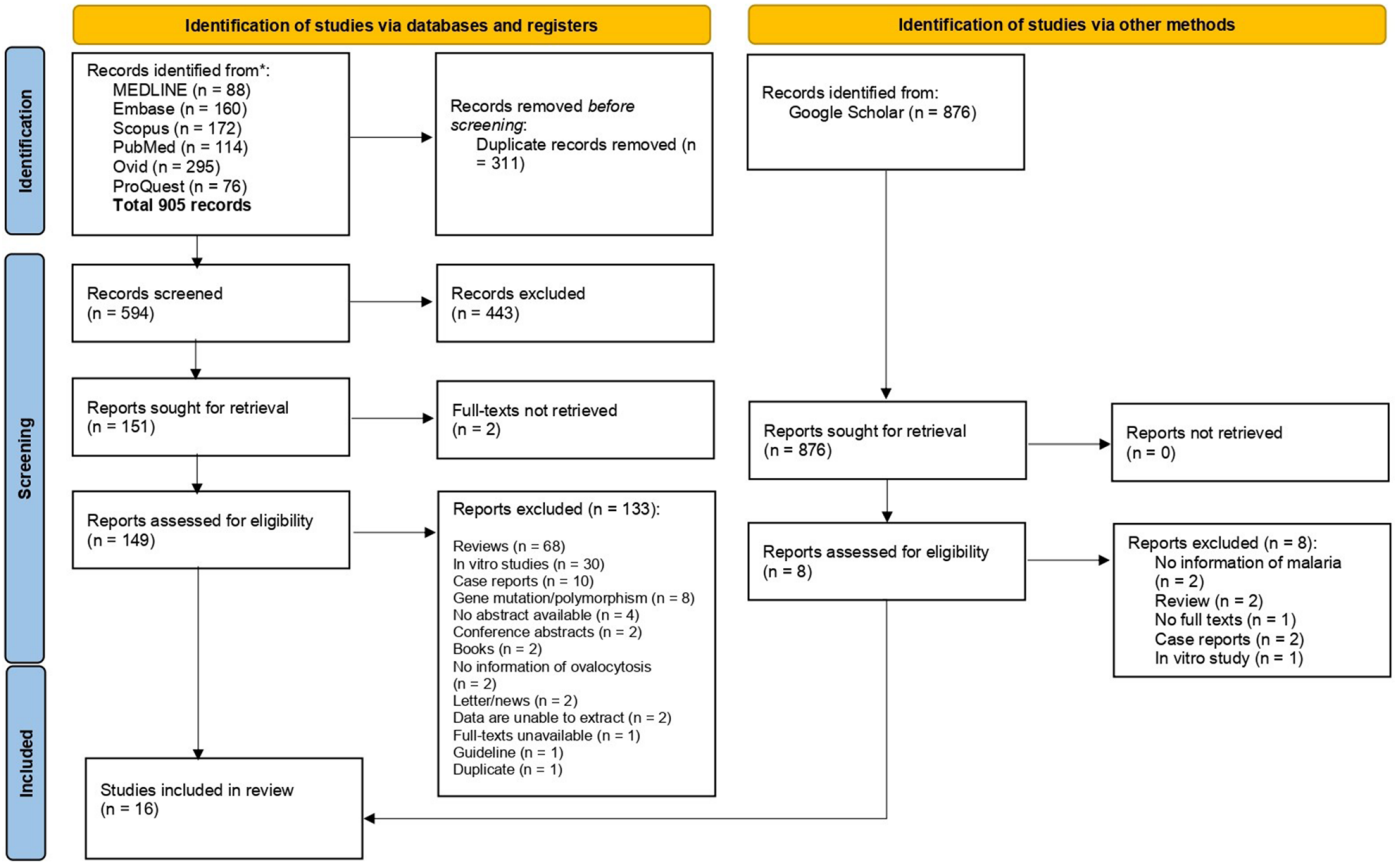
Study flow diagram.

A total of 905 articles were retrieved from our database searches: MEDLINE (n = 88), Embase (n = 160), Scopus (n = 172), PubMed (n = 114), Ovid (n = 295), and ProQuest (n = 76). After removing duplicate records (n = 311), the titles and abstracts of the remaining records were screened (n = 594) to exclude non-relevant records (n = 443). Then, the remaining records (n = 151) were assessed for full-texts and eligibility. After applying the exclusion criteria, 133 records were excluded with specific reasons: reviews (n = 68), in vitro studies (n = 30), case reports (n = 10), gene mutation or polymorphism (n = 8), no abstract available (n = 4), conference abstracts (n = 2), books (n = 2), no information on ovalocytosis (n = 2), letters or news article (n = 2), inability to extract data (n = 2), unavailability of full text (n = 1), guideline (n = 1) and duplicate article (n = 1). The searches in Google Scholar identified 876 articles, none of which met the eligibility criteria. Finally, 16 studies^15–17,26–38^ were included for qualitative and quantitative data synthesis (Fig. 1).

### General characteristics of the included studies

The included studies were published between 1977 and 2015, with the majority (43.8%) published between 2000 and 2010^16,17,28,31,32,35,37^. Study designs included cross-sectional studies (50.0%)^16,17,28,30,31,34,36,37^, case–control studies (18.8%)^26,27,29^, cohort studies (25.0%)^32,33,35,38^ and both cohort and case–control studies (6.3%)^15^. Most studies were conducted in Papua New Guinea (68.8%)^15,16,26,28,29,32–36,38^, with the remainder in Indonesia (18.8%)^17,31,37^, Malaysia (6.3%)^30^ and Thailand (6.3%)^27^. Most studies enrolled patients infected with single or mixed infections of *P. falciparum* and *P. vivax* (56.3%)^16,17,27–29,31,33,34,37^, single or mixed infections of *P. falciparum*, *P. vivax* and *P. malariae* (18.8%)^15,30,36^, and infection with *P. falciparum* only (12.5%)^26,35^. The majority of study participants were children (50.0%)^15,16,26,32–34,36,37^, followed by pregnant women (18.8%)^28,35,38^. Most of the included studies tested for SAO using a polymerase chain reaction (PCR) assay to identify band 3 deletions (75.0%)^15–17,26,28,31–35,37,38^, and the remainder investigated ovalocytosis using microscopy (25.0%)^27,29,30,36^. Malaria parasites were mainly detected using microscopy (68.8%)^16,17,26–31,34–36^, followed by a combination of microscopy with PCR (18.8%)^15,32,38^, a combination of microscopy with RDT (6.3%)^33^ or PCR only (6.3%)^37^ (Table 1). Table S2 presents details of the included studies.

**Table 1:** Characteristics of the included studies.

Characteristics	N (16 studies)	%	References
Year of publication			
2011–2015	3	18.8	^15,33,38^
2000–2010	7	43.8	^16,17,28,31,32,35,37^
Before 2000	6	37.5	^26,27,29,30,34,36^
Study designs			
Cross-sectional studies	8	50.0	^16,17,28,30,31,34,36,37^
Cohort studies	4	25.0	^32,33,35,38^
Case–control studies	3	18.8	^26,27,29^
Cohort and case–control studies	1	6.3	^15^
Country			
Papua New Guinea	11	68.8	^15,16,26,28,29,32–36,38^
Indonesia	3	18.8	^17,31,37^
Malaysia	1	6.3	^30^
Thailand	1	6.3	^27^
*Plasmodium* species			
*P. falciparum, P. vivax*	9	56.3	^16,17,27–29,31,33,34,37^
*P. falciparum, P. vivax*, *P. malariae*	3	18.8	^15,30,36^
*P. falciparum*	2	12.5	^26,35^
*P. falciparum*, *P. vivax*, *P. malariae*, *P. ovale*	1	6.3	^32^
*P. falciparum*/*P. vivax*/*P. ovale*	1	6.3	^38^
Study participants			
Children	8	50.0	^15,16,26,32–34,36,37^
Pregnant women	3	18.8	^28,35,38^
All age groups	2	12.5	^30,31^
Not specified	3	18.8	^17,27,29^
Methods for ovalocytosis detection			
SAO PCR assay for band 3 deletions	12	75.0	^15–17,26,28,31–35,37,38^
Microscopy	4	25.0	^27,29,30,36^
Methods for *Plasmodium* detection			
Microscopy	11	68.8	^16,17,26–31,34–36^
Microscopy and PCR	3	18.8	^15,32,38^
Microscopy and RDT	1	6.3	^33^
PCR	1	6.3	^37^

Abbreviations: *SAO* Southeast Asian ovalocytosis; *PCR* polymerase chain reaction; *RDT* rapid diagnostic test.

### Risk of bias

All three case–control studies^26,27,29^ and three of the cohort studies^15,32,33^ scored the maximum of 9/9 stars. Two studies^35,38^ scored 8/9 stars: one did not demonstrate the outcome of interest at the start of the study^35^ and the other lacked selection of the non-exposed cohort^38^. 'The NOS scale for case–control studies was adapted to assess the risk of bias in cross-sectional studies. Six cross-sectional studies scored 8/8 stars^16,28,30,31,34,36^, and two^17,37^ scored 7/8 stars because they lacked a ‘definition of controls’ (Table S3).

### Qualitative synthesis

Seven studies (43.8%) showed that ovalocytosis might protect against malaria infection^15,27,29,30^ or severe disease^15,26,28,36^. Apibal et al.^27^ reported that patients with malaria had an increased percentage of ovalocytes compared with uninfected patients (*P. falciparum*: mean, 6.3 cells; *P. vivax*: mean, 8.3 cells; normal individuals: mean, 0.6 cells). Benet et al.^28^ reported that severe malaria infections in placental tissues were less common in pregnant women with SAO than in pregnant women in the control group. Cattani et al.^29^ reported that patients with ovalocytosis had a lower infection rate of *P. falciparum* (*P* = 0.044), *P. vivax* (*P* = 0.009) and all species of malaria combined (*P* = 0.013) compared with those without ovalocytosis. Foo et al.^30^ demonstrated fewer ever-positive ovalocytes for *P. falciparum* (*P* < 0.05) or any parasite species (*P* < 0.05) compared with the controls. Additionally, malaria parasitaemia was lower in individuals with ovalocytes compared with the controls. Rosanas-Urgell et al.^15^ reported that SAO was associated with a statistically significant reduction of 46% in the incidence of clinical *P. vivax* episodes and a 52% reduction in *P. vivax* blood-stage reinfection. Furthermore, in the case–control study, SAO was associated with protection against severe *P. falciparum* malaria (OR = 0.38, *P* = 0.014) but not with protection against uncomplicated *P. falciparum* malaria. Allen et al.^26^ reported the absence of SAO band 3 in 68/68 (100%) of children with cerebral malaria compared with 6/68 (8.8%) matched community controls (OR = 0, 95% CI 0.0–0.85). Serjeantson et al.^36^ suggested that patients with ovalocytosis were more resistant to severe malaria than those with normocytes because the ratio of parasitaemia in 112 children with ovalocytes compared with 741 children with normocytes was 1.05 for *P. falciparum*; 0.90 for *P. vivax*; 0.54 for *P. malariae*, and 0.91 for infection with any *Plasmodium* species.

Eight studies (8/16, 50%) showed no association of SAO with malaria infection or severity^16,17,31,32,34,35,37,38^. Fowkes et al.^16^ found no association of SAO with *P. falciparum* prevalence (*P* = 0.29) or with mean parasite density (*P* = 0.66). Kimura et al.^17^ found no difference in the prevalence of SAO between patients with malaria and controls (*P* > 0.8) and no difference in the frequency of SAO between patients with *P. falciparum* and *P. vivax* malaria. Although Kimura et al.^31^ discovered a link between a higher rate of ovalocytes and a lower risk of malaria infection, they found that SAO did not protect against malaria infection. Lin et al.^32^ showed no significant associations between *P. falciparum* infection and SAO (*P* > 0.2). O’Donnell et al.^34^ demonstrated no difference in the proportion of SAO in acute malaria and the community controls (8.8% vs 6.6%, *P* = 0.57). However, the degree of ovalocytosis was significantly lower in children with SAO during acute malaria compared with the community controls (*P* = 0.025). O’Donnell et al.^35^ showed that the SAO genotype was not associated with the frequency of placental *P. falciparum* infection (placental parasitaemia with a normal genotype: 24.5%, placental parasitaemia with SAO: 21.1%). Shimizu et al.^37^ reported no significant difference in the prevalence of asymptomatic malaria infection between participants with or without SAO (*P* > 0.05), and Stanisic et al.^38^ reported no significant difference in the frequency of malaria infection among participants with or without SAO (*P* = 0.62). Manning et al.^33^ reported a frequently higher proportion of ovalocytosis in *P. falciparum* (5.7%) than *P. vivax* (3.8%) and mixed infections (4.3%).

### Risk of malaria among participants with ovalocytosis

**Figure 2:**
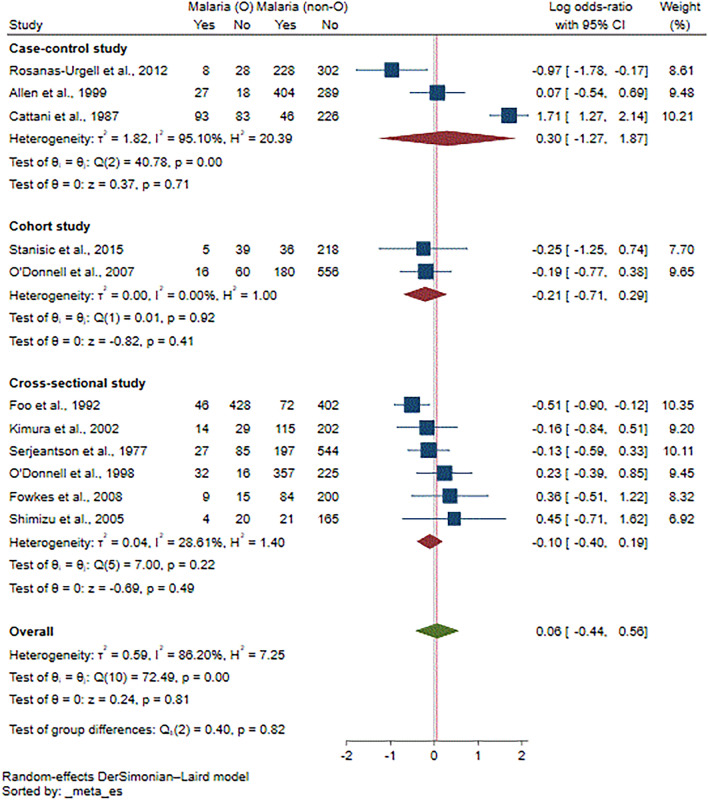
Forest plot showing the pooled log odds ratio (OR) of the association between Southeast Asian ovalocytosis (SAO) and malaria infection. Abbreviations: *Malaria (O)* malaria infection in SAO RBCs; *Malaria (non-O)* malaria infection in non-SAO RBCs; *CI* confidence interval; *blue square* effect estimate (log OR); *crimson diamond* pooled log OR in each subgroup; *green diamond* pooled log OR in all included studies.

**Figure 3:**
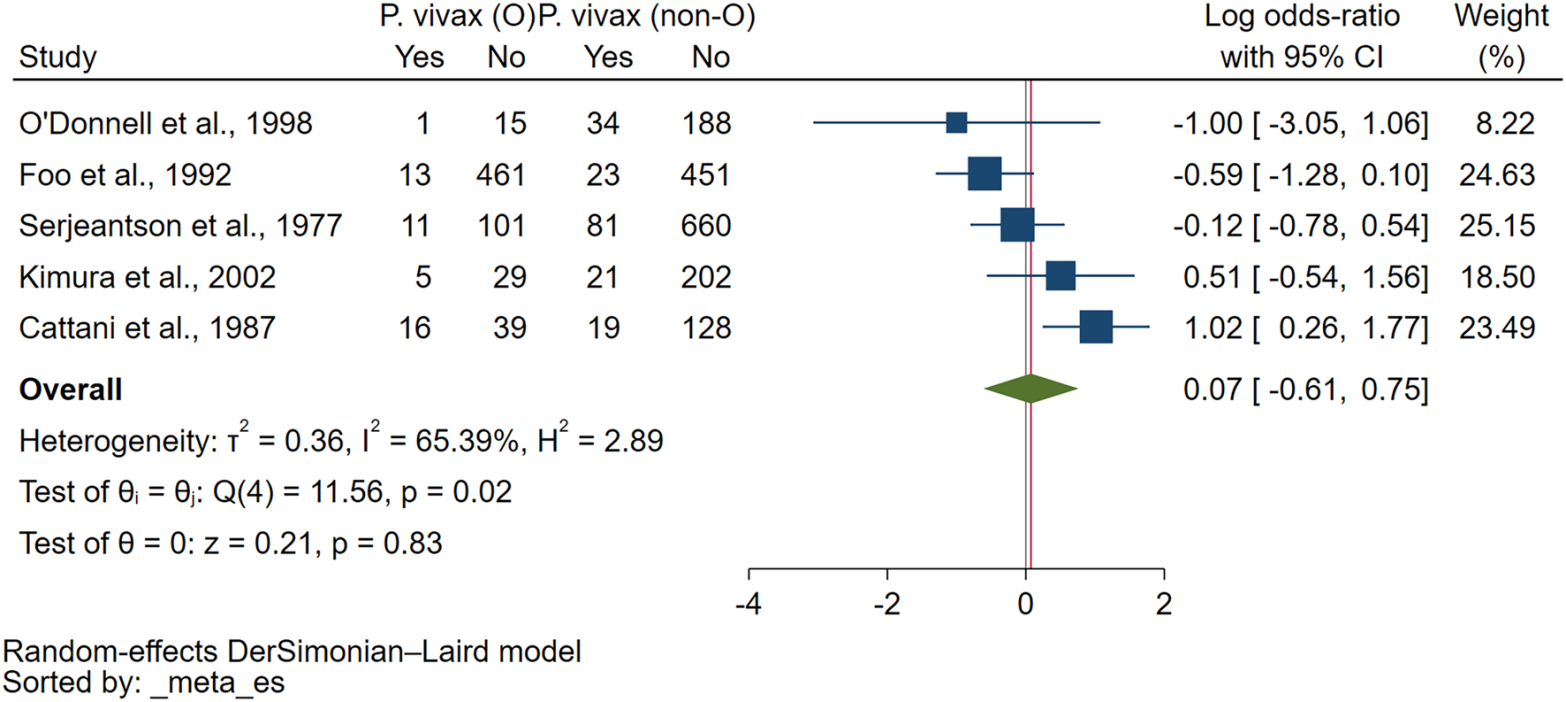
Forest plot showing the pooled log odds ratio (OR) of the association between Southeast Asian ovalocytosis (SAO) and *P. vivax* infection. Abbreviations: *P. vivax* (O), *P. vivax* infection in SAO RBCs; *P. vivax* (non-O), *P. vivax* infection in non-SAO RBCs; *CI* confidence interval; *blue square* effect estimate (log OR); *green diamond*, pooled log OR in all included studies.

Eleven studies^15–17,26,29,30,34–38^ were included in the meta-analysis of the risk of malaria among participants with ovalocytosis. Overall, the results demonstrated no difference in the log OR of malaria between patients with and without ovalocytosis (*P* = 0.81, log OR = 0.06, 95% CI − 0.44 to 0.56, I^2^: 86.20%; 11 studies; Fig. 2). The meta-regression analyses using study design, country, participants’ group, *Plasmodium* species, method used to investigate ovalocytosis and method for malaria detection as covariates showed that none of these impacted the pooled effect estimate. Thus, the subgroup analysis was discontinued (Table S4). Since Rosanas-Urgell et al.^15^ showed that SAO was associated with a statistically significant reduction in the incidence of *P. vivax* infection, the meta-analysis was refined to *P. vivax* exclusively to prevent any interference from a concomitant *P. falciparum* infection. However, the results showed no difference in the log OR of *P. vivax* infection between patients with and without ovalocytosis (*P* = 0.83, log OR = 0.07, 95% CI: − 0.61 to 0.75, I^2^: 65.39%; 5 studies; Fig. 3).

### Sensitivity analysis

**Figure 4:**
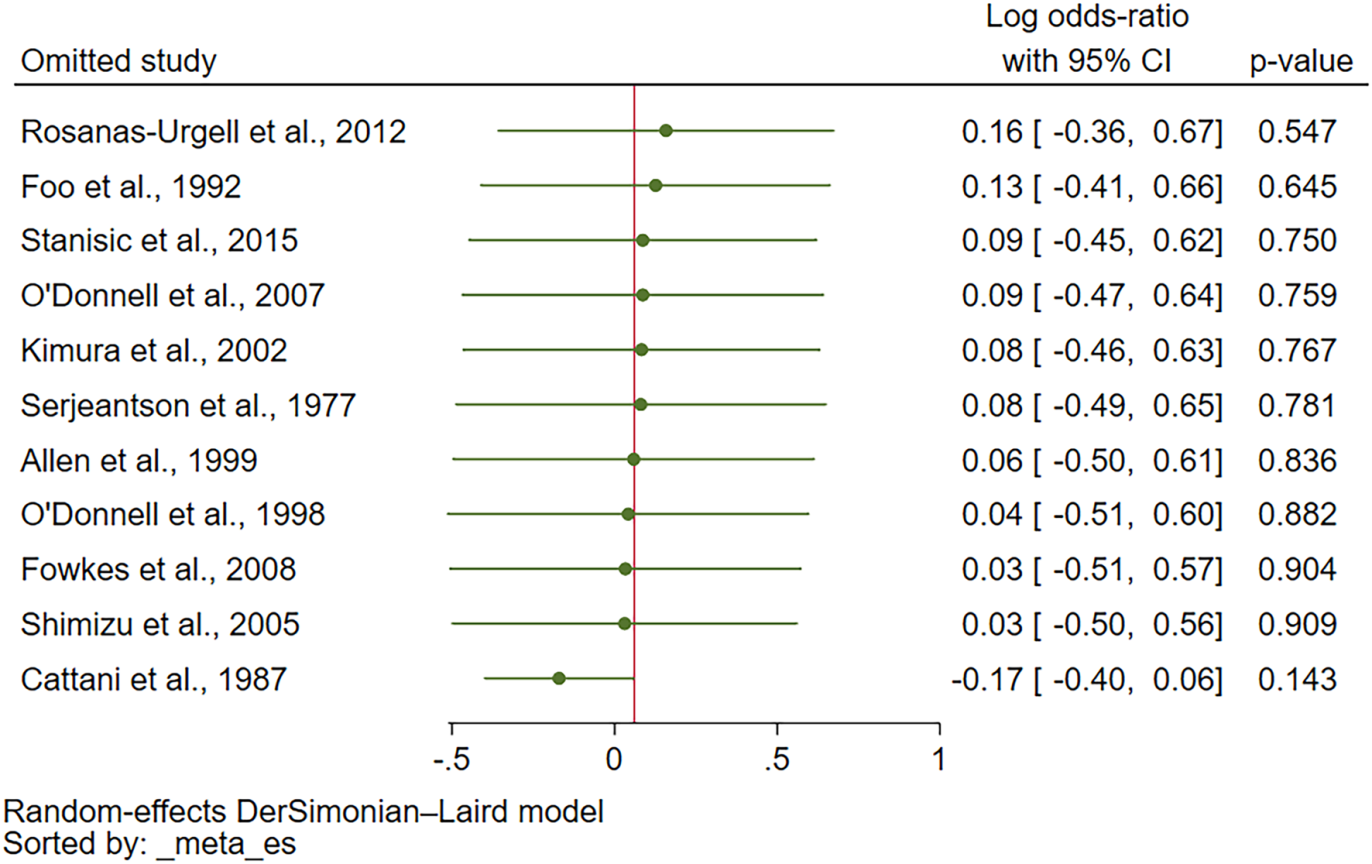
Leave-one-out sensitivity analysis to identify outliers in the meta-analysis of the log odds ratio between Southeast Asian ovalocytosis and malaria infection. Abbreviations: *CI* confidence interval; *green dot* effect estimate.

**Figure 5:**
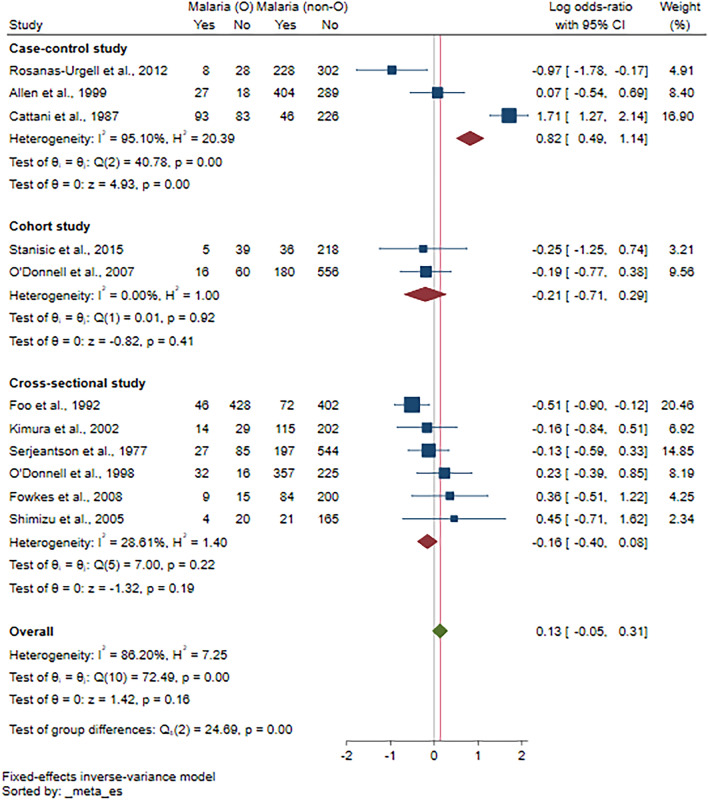
Sensitivity analysis using the fixed-effects model for the pooled log odds ratio between Southeast Asian ovalocytosis and malaria infection. Abbreviations: *CI* confidence interval; *blue square* effect estimate (log OR); *crimson diamond* pooled log OR in each subgroup; *green diamond* pooled log OR in all included studies.

The leave-one-out sensitivity analysis did not identify any outliers in the meta-analysis (*P* values in re-run analyses > 0.05; Fig. 4), indicating the robustness of the results. Meta-analysis using fixed-effect models was performed to test whether the difference in statistical models affected the pooled effect estimate. The results showed a decreased log OR of malaria among patients with ovalocytosis compared with those without ovalocytosis (*P* < 0.01, log OR = 0.82, 95% CI 0.49–1.14, I^2^: 95.10%; 3 studies; Fig. 5).

### Publication bias

**Figure 6:**
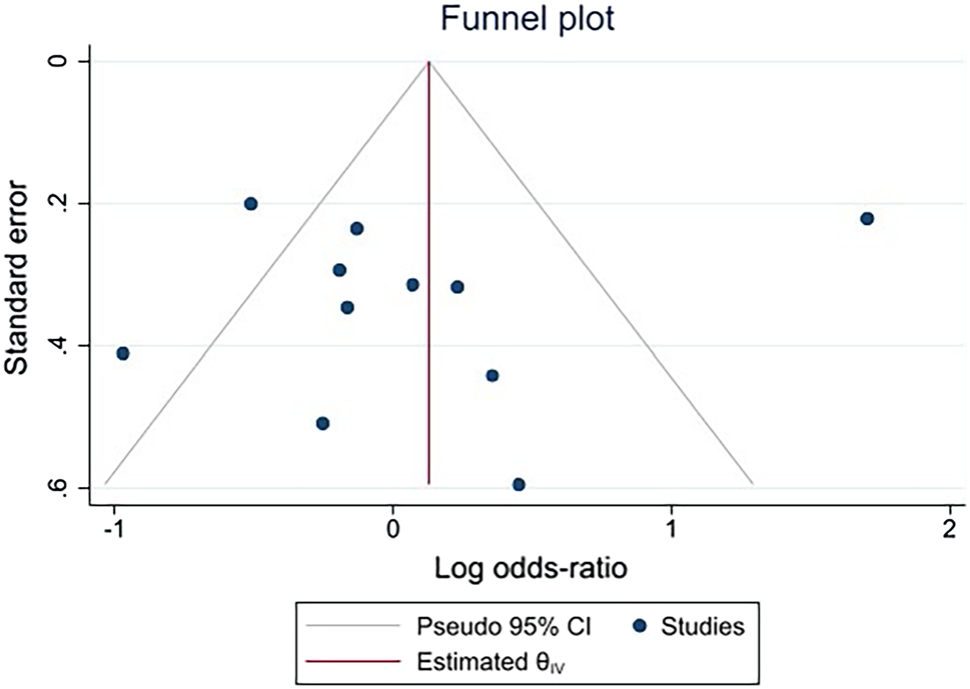
Funnel plot demonstrating the asymmetrical distribution of the log odds ratio of individual studies.

Publication bias was assessed using a funnel plot and Egger’s test. The funnel plot showed the asymmetrical distribution of the log OR and indicated the standard error of the log OR (Fig. 6). Egger’s test demonstrated that the small-study effect was not significant (*P* = 0.68). The results of both tests indicated a publication bias among the included studies. Although the trim-and-fill method was applied to correct for publication bias, the subsequent results showed no difference in the log OR of malaria among patients with ovalocytosis compared with those without ovalocytosis (log OR = 0.297, 95% CI − 0.126 to 0.721).

## Discussion

SAO is a disorder of the RBC membrane caused by a mutation in the gene for band 3 protein, which leads to the formation of unique linear oligomers in the RBC membrane. Our meta-analysis results demonstrated no association between SAO and malaria infection, and almost all of the included studies demonstrated no association between SAO and malaria infection^16,17,26,34–38^. Meanwhile, studies by Rosanas-Urgell et al.^15^ and Foo et al.^30^ both showed lower odds of malaria infection in SAO. Conversely, only the study by Cattani et al.^29^ reported higher odds of malaria infection in SAO. According to Rosanas-Urgell et al.^15^, SAO provides partial protection against *P. vivax* but not against *P. falciparum* infection or uncomplicated disease. Nevertheless, our meta-analysis contradicted these results, with no difference in the risk of *P. vivax* infection between patients with and without ovalocytosis. These findings suggest that ovalocytosis may not be a protective factor against *P. vivax* infection, at least for the limited studies included in this meta-analysis.

The protective mechanism of SAO against malaria is unknown, and several theories have been proposed. Firstly, modification of the RBC membrane may affect the ability of merozoites to invade RBCs and prevent the development of parasites within RBCs^15^. Secondly, alterations in RBC structures may impair the binding of malarial proteins from the parasite to the RBC membrane, thereby inhibiting parasite growth within RBCs^39^. Thirdly, SAO RBCs may provide protection against cerebral malaria due to the redistribution of sequestered infected RBCs away from the brain^32^. Similar to the protection against cerebral malaria, SAO RBCs may prevent parasitised RBCs from adhering to receptors in the placenta, thereby protecting against placental malaria. However, a study on placental samples of pregnant women demonstrated that SAO RBCs did not reduce the incidence of placental malaria and, therefore, did not protect against placental malaria^35^. Another study on pregnant women revealed that primigravidae with SAO had a lower prevalence of placental infection than primigravidae without SAO, whereas there was no difference in the prevalence and severity of placental infections between SAO and non-SAO multigravidae, indicating that the gravidae status in women with SAO confounds the association between SAO and placental infection. Thus, the mechanism by which SAO RBCs protect against placental malaria among primigravidae was suggested to be non-immune- rather than immune-based^28^. Since SAO RBCs protect against cerebral malaria or severe diseases but not uncomplicated diseases, this suggests that the protection involves post-invasion mechanisms, such as preventing sequestration of infected RBCs^26,40,41^.

Our systematic review showed heterogeneous associations between SAO and malaria in individual studies. Most studies demonstrated no association of SAO with malaria infections or severity^16,17,31,32,34,35,37,38^. However, some showed that ovalocytosis might protect against malaria infection^15,27,29,30^ or severe disease^15,26,28,36^. Nevertheless, our meta-analysis results demonstrated no association between SAO and malaria infection. These results were stratified according to the study design, without or with minimal heterogeneity in cohort studies^35,38^ and in cross-sectional studies^16,17,30,34,36,37^. High or substantial heterogeneity was found in three case–control studies^15,26,29^. Besides differences in study design, the controversial association of SAO in the literature was suggested to be caused by the presence of molecularly heterogeneous SAO because SAO without B3∆27 was associated with decreased malaria infection rates^31^. Additionally, another study indicated that the inconsistency of SAO-related protection against malaria might be due to the detection of SAO with low sensitivity^34^.

Our systematic review and meta-analysis had several limitations. Firstly, the number of studies included in the meta-analysis was limited, which might limit the interpretation of the results. Nonetheless, because the results of the studies included in the meta-analysis were consistent, the findings were sufficiently robust to support the conclusion that there is no link between SAO and malaria infection. Secondly, a meta-analysis of the odds of severe disease between severe and non-severe diseases could not be performed due to the limited number of studies reporting an association between SAO and malaria severity. Third, there was heterogeneity in the association between ovalocytosis and malaria infection between studies. Although we conducted a meta-regression analysis, no probable source of heterogeneity was found. Fourth, although our meta-analysis revealed that ovalocytosis did not protect against *P. vivax* malaria, some studies have suggested that SAO mainly protects against *P. vivax* malaria. Thus, it would be interesting for further studies to refine the investigation to that of *P. vivax* exclusively to assess the extent of this protection without any interference from a concomitant *P. falciparum* infection.

## Conclusion

Our systematic review revealed the heterogeneity of individual studies regarding the association between SAO and malaria. However, our meta-analysis results demonstrated no protection of SAO RBCs against malaria infection. Hence, the association of SAO with malaria infection or disease severity should be further investigated by larger studies.

## Supplementary Information

Supplementary Information 1.

Supplementary Information 2.

Supplementary Table S1.

Supplementary Table S2.

Supplementary Table S3.

Supplementary Table S4.

## Data Availability

All data relating to the present study are available in this manuscript and supplementary files.
