# Supplementary material for: Association between ovalocytosis and Plasmodium infection: a systematic review and meta-analysis
Source: Sci Rep. 2023 May 3;13:7164. doi: 10.1038/s41598-023-34170-3 (PMC10156661; doi:10.1038/s41598-023-34170-3)
Supplement: Supplementary file 3 — Supplementary Table S1. [file 41598_2023_34170_MOESM3_ESM.docx]

**Association between ovalocytosis and *Plasmodium* infection: A systematic review and meta-analysis**

Kwuntida Uthaisar Kotepui^1^, Aongart Mahittikorn^2*^, Frederick Ramirez Masangkay^3^ & Manas Kotepui^1*^

^1^ Medical Technology, School of Allied Health Sciences, Walailak University, Tha Sala, Nakhon Si Thammarat, Thailand

^2^Department of Protozoology, Faculty of Tropical Medicine, Mahidol University, Bangkok, Thailand

^3^Department of Medical Technology, University of Santo Tomas, Manila, Philippines

**^*^Corresponding author**

Manas Kotepui: manas.ko@wu.ac.th, Tel.: +66954392469

Kwuntida Uthaisar Kotepui: [kwuntida.ut@wu.ac.th](mailto:kwuntida.ut@wu.ac.th)

Aongart Mahittikorn: aongart.mah@mahidol.ac.th

Frederick Ramirez Masangkay: [frederick_masangkay2002@yahoo.com](mailto:frederick_masangkay2002@yahoo.com)

**Table S1. Search strategy**

**PubMed**

**30 December 2022**

| **No.** | **Query** | **Results** |
| --- | --- | --- |
| 3 | #1 AND #2 | 114 |
| 2 | (((malaria) OR (malaria[MeSH Terms])) OR (Plasmodium)) OR (Plasmodium[MeSH Terms]) | 120,307 |
| 1 | (((Ovalocytosis) OR (Ovalocytosis[MeSH Terms])) OR (Elliptocytosis)) OR (Elliptocytosis[MeSH Terms]) | 1,002 |

**Scopus**

**30 December 2022**

| **No.** | **Query** | **Results** |
| --- | --- | --- |
| 3 | 1 AND 2 | 172 |
| 2 | (malaria OR plasmodium OR "remittent fever" OR "marsh fever" OR paludism ) | 153,836 |
| 1 | TITLE-ABS-KEY ( elliptocyt* OR ovalocyt* ) | 1,461 |

**MEDLINE**

| **No.** | **Search terms/Search strategy** | **Date** |
| --- | --- | --- |
| 1 | (Elliptocyt* OR Ovalocyt*) AND (malaria OR Plasmodium OR "Remittent Fever" OR "Marsh Fever" OR Paludism)  Search results: 80 | **30 December 2022** |

**Embase**

| **No.** | **Search terms/Search strategy** | **Date** |
| --- | --- | --- |
| 1 | (Elliptocyt* OR Ovalocyt*) AND (malaria OR Plasmodium OR "Remittent Fever" OR "Marsh Fever" OR Paludism)  Search results: 160 | **30 December 2022** |

**ProQuest**

| **No.** | **Search terms/Search strategy** | **Date** |
| --- | --- | --- |
| 1 | (Elliptocyt* OR Ovalocyt*) AND (malaria OR Plasmodium OR "Remittent Fever" OR "Marsh Fever" OR Paludism)  Search results: 76 | **30 December 2022** |
