# Supplementary material for: Association between ovalocytosis and Plasmodium infection: a systematic review and meta-analysis
Source: Sci Rep. 2023 May 3;13:7164. doi: 10.1038/s41598-023-34170-3 (PMC10156661; doi:10.1038/s41598-023-34170-3)
Supplement: Supplementary file 6 — Supplementary Table S4. [file 41598_2023_34170_MOESM6_ESM.docx]

**Association between ovalocytosis and *Plasmodium* infection: A systematic review and meta-analysis**

Kwuntida Uthaisar Kotepui^1^, Aongart Mahittikorn^2*^, Frederick Ramirez Masangkay^3^ & Manas Kotepui^1*^

^1^ Medical Technology, School of Allied Health Sciences, Walailak University, Tha Sala, Nakhon Si Thammarat, Thailand

^2^Department of Protozoology, Faculty of Tropical Medicine, Mahidol University, Bangkok, Thailand

^3^Department of Medical Technology, University of Santo Tomas, Manila, Philippines

**^*^Corresponding author**

Manas Kotepui: manas.ko@wu.ac.th, Tel.: +66954392469

Kwuntida Uthaisar Kotepui: [kwuntida.ut@wu.ac.th](mailto:kwuntida.ut@wu.ac.th)

Aongart Mahittikorn: aongart.mah@mahidol.ac.th

Frederick Ramirez Masangkay: [frederick_masangkay2002@yahoo.com](mailto:frederick_masangkay2002@yahoo.com)

**Table S4. Meta-regression results**

| **Parameters** | **P value** | **R-squared (%)** | **tau2** | **I^2^ (%)** | **Number of studies** |
| --- | --- | --- | --- | --- | --- |
| Study design | 0.70 | 15.41 | 0.50 | 83.26 | 11 |
| Country | 0.77 | 0 | 0.67 | 86.35 | 11 |
| Participants ‘group | 0.27 | 37.73 | 0.37 | 75.37 | 11 |
| *Plasmodium* spp. | 0.84 | 0.36 | 0.80 | 89.07 | 11 |
| Method for investigate the ovalocytosis | 0.47 | 0 | 0.65 | 86.75 | 11 |
| Method for malaria detection | 0.47 | 0 | 0.59 | 87.73 | 11 |
